# Supplementary material for: Key Care Provision Aspects That Affect Care Transition in the Long-Term Care Systems: Preliminary Review Findings
Source: Int J Environ Res Public Health. 2022 May 24;19(11):6402. doi: 10.3390/ijerph19116402 (PMC9180334; doi:10.3390/ijerph19116402)
Supplement: Supplementary file 1 [file ijerph-19-06402-s001.zip › ijerph-1727051-suppl/Supplementary File S2_EW_Search_Strategy.pdf]

Supplementary File S2

Figure. Stages of the selection process

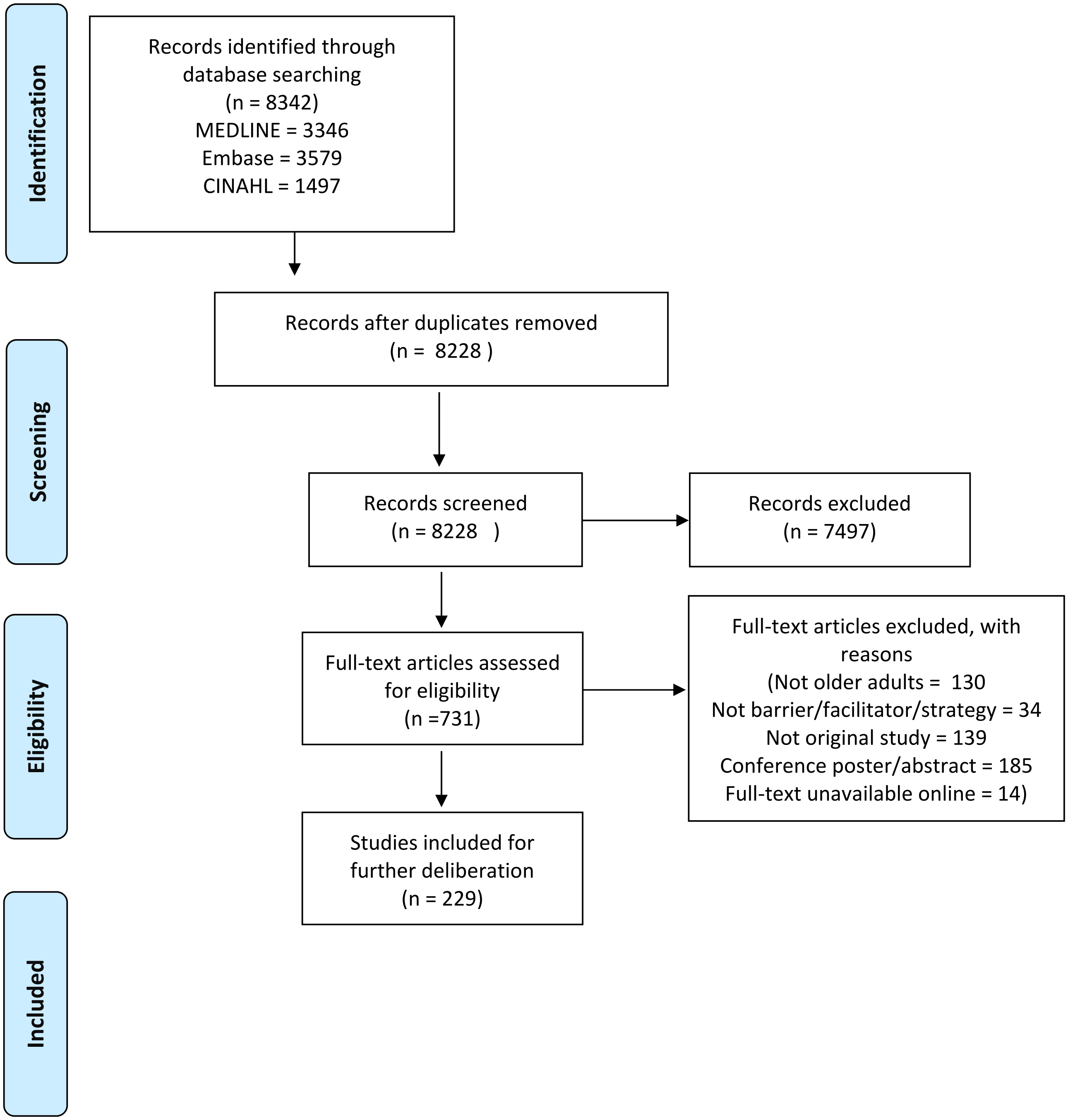

Based on:. Moher D, Liberati A, Tetzlaff J, Altman D, The PRISMA Group. Preferred Reporting Items for Systematic Reviews and Meta-Analyses: The PRISMA Statement. PLoS Medicine. 2009;6(7).
