# Supplementary material for: Key Care Provision Aspects That Affect Care Transition in the Long-Term Care Systems: Preliminary Review Findings
Source: Int J Environ Res Public Health. 2022 May 24;19(11):6402. doi: 10.3390/ijerph19116402 (PMC9180334; doi:10.3390/ijerph19116402)
Supplement: Supplementary file 1 [file ijerph-19-06402-s001.zip › ijerph-1727051-suppl/Supplementary File S3_EW.pdf]

Supplementary File S3

Table S2. Publications per year per category*

	**Year**																
**Category**	**2005**	**2006**	**2007**	**2008**	**2009**	**2010**	**2011**	**2012**	**2013**	**2014**	**2015**	**2016**	**2017**	**2018**	**2019**	**2020**	**Total**
**Organizational - General**																	**270****
Coordination of resources	2	3	7	2	2	2	3	7	7	9	6	7	10	14	9*	*	**90**
Transfer of information and care responsibility	4	0	4	2	3	3	7	4	2	4	2	5	4	7	0*	*	**51**
Communication of health professionals	2	2	2	1	2	1	4	1	7	2	3	2	4	2	1*	*	**36**
Training and education of staff	3	2	2	4	3	3	5	4	4	3	1	4	6	6	0*	*	**24**
Opinions health professionals	1	1	1	0	1	0	3	2	2	2	1	4	1	1	2*	*	**22**
Education and involvement of the patient and family	0	0	1	0	1	2	3	1	2	2	2	1	0	2	1*	*	**18**
Opinions patients and family	1	0	1	1	0	0	1	2	3	0	2	1	2	2	1*	*	**17**
E-health	0	1	1	0	0	1	1	2	1	1	1	1	2	0	0*	*	**12**
**Organizational – Disease/Condition Specific**																	**33****
Coordination of resources	1	0	0	1	0	1	1	0	1	4	2	1	0	1	0*	*	**12**
Education and involvement of the patient and family	0	0	0	0	0	1	1	0	1	1	0	1	0	0	0*	*	**5**
Opinions health professionals	0	0	0	0	0	0	0	1	0	0	1	0	0	2	0*	*	**4**
Opinions patients and family	0	2	0	0	0	0	0	0	1	0	0	0	1	0	0*	*	**4**
Communication of health professionals	0	0	0	0	0	1	1	0	0	0	0	0	0	1	0*	*	**3**
Training and education of staff	0	0	0	0	0	0	0	0	0	0	1	1	0	0	0*	*	**2**
E-health	0	0	0	0	0	1	0	0	0	0	0	0	1	0	0*	*	**2**
Transfer of information and care responsibility	0	0	0	0	0	0	1	0	0	0	0	0	0	0	0*	*	**1**
**Financial aspects**																	
Provider payment mechanisms/ rewards/ penalties	1	0	1	0	0	1	2	0	1	2	2	2	3	1	*	*	**16**
**Social aspects**																	
**Social aspects**	2	3	1	1	0	0	1	2	0	0	0	3	2	3	2*	*	**18**

*Search was conducted in March 2020. Therefore, the list of publications for year 2019 and 2020 may be incomplete.

**Some publications were mentioned in more than one category (for example they discussed coordination of resources and education and involvement of the patient and family)
