# Supplementary material for: Key Care Provision Aspects That Affect Care Transition in the Long-Term Care Systems: Preliminary Review Findings
Source: Int J Environ Res Public Health. 2022 May 24;19(11):6402. doi: 10.3390/ijerph19116402 (PMC9180334; doi:10.3390/ijerph19116402)
Supplement: Supplementary file 1 [file ijerph-19-06402-s001.zip › ijerph-1727051-suppl/Supplementary File S1_EW_Protocol.pdf]

Supplementary File S1

Review protocol

The review protocol is registered in the International Prospective Register of Systematic Reviews (PROSPERO) under identification number CRD42020162566. This review was performed as part of the European TRANS-SENIOR project. The dual focus of the TRANS-SENIOR research is on avoiding unnecessary care transitions and improving care for transitions that are needed.

The objective of the systematic review was to identify all studies that address the financial and/or organizational aspects of care transition in the LTC systems. We perform the search in a systematic way to minimize the potential bias. Specifically, the PRISMA-P (Preferred Reporting Items for Systematic Reviews and Meta-Analyses) guidelines were followed to design the search strategy.

Search strategy

The search was conducted in MEDLINE, Embase and CINAHL. The search strategy was developed in consultation with an academic health sciences librarian. Given the search objective, three components were used to build the search terms for the identification of key financial and organizational aspects affecting care transition in LTC systems. These components included: (1) old or geriatric or senior; (2) care transition or coordinated care or care continuity; (3) financing or organization. Moreover, different forms of the above words as well as relevant synonyms and subject heading terms appropriate for each database, were taken into account. All search terms can be found in Table S1.

Table S1. Search terms

Category 1	Category 2	Category 3
Elderly	Patient*	Financ*
Aged	Care*	Organi*
Aging	Clinical handover	Purchas*
Old	Coordinated care	Funding
Senior	Coordination of care	Provision
Geriatric	Continuity of care	Reimbursement
	Integrated care	

Patient* captures “patient handover”, “patient transfer”, “patient discharge” etc. Care* captures “care coordination”, “care continuity”, “ care continuum” etc. Financ* captures “financing”, “financial” etc. Organi* captures “organizational”, “organizing”, “organization” etc. Purchas* captures “purchasing”, “purchase” etc.

The exact chain of keywords for different databases is presented below.

EMBASE

'aged'/exp OR aged:ab, ti OR aging:ab, ti OR elderly:ab, ti OR old:ab, ti OR senior*:ab, ti OR geriatric:ab, ti AND 'patient handoff' OR 'patient handover' OR 'hospital discharge'/exp OR 'patient transfer' OR 'transitional care'/exp OR 'clinical handover'/exp OR 'coordinated care'/exp OR 'coordination of care' OR 'care coordination' OR 'integrated care' OR 'patient care'/exp OR 'care continuum' AND 'financial management'/exp OR 'organization'/exp OR 'provision' OR 'purchasing'/exp OR 'reimbursement'/exp

MEDLINE

(“aged” [MeSH Terms] OR “aged” [Title/Abstract] OR “aging” [Title/Abstract] OR “elderly” [Title/Abstract] OR “old” [Title/Abstract] OR “senior*” [Title/Abstract] OR “geriatric” [Title/Abstract]) AND (“patient handoff” [MeSH Terms] or “patient handoff” [All Fields] OR “patient handover” [All Fields] OR “patient discharge” [MeSH Terms] OR “patient discharge” [All Fields] OR “patient transfer” [MeSH Terms] OR “Patient transfer” [All Fields] OR “transitional care” [MeSH Terms] OR “transitional care” [All Fields] OR “clinical handover” [All Fields] OR “coordinated care” [All Fields] OR “coordination of care” [All Fields] OR “care coordination” [All Fields] OR “integrated care” [All Fields] OR “care continuity” [All Fields] OR “continuity of care” [All Fields] OR “care continuum” [All Fields]) AND (“financing” [All Fields] OR “financ*” [All Fields] OR "funding" [All Fields] OR “organised” [All Fields] OR “organized” [All Fields] OR “organisational” [All Fields] OR “organizational” [All Fields] OR “organizing” [All Fields] OR “organising” [All Fields] OR “organization” [All Fields] OR “organisation” [All Fields] OR “provision” [All Fields] OR purchasing [All Fields] OR purchase* [All Fields] OR “reimbursement” [All Fields])

CINAHL
((MM "aged") OR (TI "aged") OR (AB "aged") OR (TI "aging") OR (AB "aging") OR (TI "elderly") (AB "elderly") OR (TI "old") OR (AB "old") OR (TI "senior*") OR (AB "senior*") OR (TI "geriatric") OR (AB "geriatric")) AND ((MM "hand off") OR (TX "hand off") OR (TX "patient handover") OR (MM "patient discharge") OR (TX "patient discharge") OR (TX "patient transfer") OR (MM "transitional care") OR (TX "transitional care") OR (TX "clinical handover") OR (TX "coordinated care") OR (TX "coordination of care") OR (TX "care coordination") OR (TX "integrated care") OR (MM "continuity of patient care") OR (TX "continuity of patient care") OR (TX "care continuum")) AND ((MM "financing, organized") OR (TX "financing") OR (TX "financ*") OR (TX "organi*ed") OR (TX "organi*ational") OR (TX "organi*ing") OR (TX "organi*ation") OR (TX "provision") OR (TX "purchasing") OR (TX "purchase*") OR (MM "reimbursement mechanism") OR (MM "reimbursement mechanism") OR (TX "reimbursement mechanism") OR (TX "reimbursement"))

The search was limited to literature published between March 2005 and March 2020 (the last 15 years). No geographical or language restrictions were implied.

Eligibility criteria

The search included studies that focus on transitional care between the settings among older adults 60+. No restrictions were placed on participants' gender or other demographic characteristics. All primary epidemiological observational study designs (i.e., cross-sectional, cohort, case-control studies) ecological studies and experimental studies were eligible. Reviews, commentaries, editorials and other non-primary research articles were not eligible. Moreover, studies were included if (a) they reported on financial and organizational aspects of care transition in the long-term care systems, (b) reported on financial and organizational aspects of care transition at the macro-level, mainly focusing on transitions between different settings and not within the setting (c) and their focus was on older adults (60 years or older). Studies were also included if data stratification was performed for individuals aged 60+. Studies were excluded if (a) they reported on financial and organizational aspects of care transition at the micro-level, care transition within the setting, (b) focus of the study was on individuals younger than 60 years of age, (c) focus was on palliative, hospice or end-of-life care.

Study selection

The selection process, based on the above inclusion and exclusion criteria, had three phases. First, a screening based on title and abstract was performed to identify potentially relevant studies, where 10% of the excluded papers were independently reviewed by a second reviewer (one of the co-authors). This was followed by a second screening based on full text to confirm the relevance of the studies. Third, the reference lists of the selected studies were screened to check for additional studies. Any disagreement about the eligibility of studies was resolved through discussion and consensus among all co-authors.

Data extraction and management

A data extraction form will be developed and pre-tested. The extracted information will include among others: study country; study methodology; study setting; study population; details of the intervention and control (if applicable); outcome measures related to care transition and timing; study limitations; and information for the assessment of risk of bias; study main conclusions; identified transitional care challenges; and proposed improvement strategies.

Quality assessment

The methodological quality and risk of bias of studies included in the review will be rigorously appraised with the use of Quality Assessment Tool for Quantitative Studies developed by Effective Public Health Practice Project and Critical Appraisal Skills Programme for qualitative studies. These tools enable the researcher to rank each study according to the guidelines provided along with the tools. Based on the score, each study will be classified either as low, average or high-quality study.

Data synthesis and analysis

The method of directed (relational) content analysis by Hsieh and Shannon will be applied to perform analysis of the publications. Within this approach, we will identify the categories (themes) relevant to the review objective. The preliminary literature results provided us with the guidance for initial codes. Thus, for the purpose of the review following themes will be used (see also Figure 2):

Themes for the review on organizational aspects of care transition:
- general organizational aspects that affect care transition
- disease-specific organizational aspects that affect care transition

Themes for the review on financial aspects of care transition (see also Figure 2):
- provider payment mechanisms
- rewards
- penalties

Based on these themes, the data extraction will be performer. Review results will be presented per themes in a narrative manner. Additionally, the results will be presented in the form of descriptive tables.
